# Lipidomics of phospholipase A_2_ reveals exquisite specificity in macrophages

**DOI:** 10.1016/j.jlr.2024.100571

**Published:** 2024-05-23

**Authors:** Gosia M. Murawska, Aaron M. Armando, Edward A. Dennis

**Affiliations:** Department of Chemistry and Biochemistry and Department of Pharmacology, School of Medicine, University of California at San Diego, La Jolla, CA, USA

**Keywords:** cPLA_2_, sPLA_2_, iPLA_2_, LpPLA_2_, PAFAH, RAW264.7 cells, lipidomics, macrophages, inflammation

## Abstract

Phospholipase A_2_ (PLA_2_) constitutes a superfamily of enzymes that hydrolyze phospholipids at their *sn*-2 fatty acyl position. Our laboratory has demonstrated that PLA_2_ enzymes regulate membrane remodeling and cell signaling by their specificity toward their phospholipid substrates at the molecular level. Recent *in vitro* studies show that each type of PLA_2_, including Group IVA cytosolic PLA_2_ (cPLA_2_), Group V secreted PLA_2_ (sPLA_2_), Group VIA calcium independent PLA_2_ (iPLA_2_) and Group VIIA lipoprotein-associated PLA_2_, also known as platelet-activating factor acetyl hydrolase, can discriminate exquisitely between fatty acids at the *sn*-2 position. Thus, these enzymes regulate the production of diverse PUFA precursors of inflammatory metabolites. We now determined PLA_2_ specificity in macrophage cells grown in cell culture, where the amounts and localization of the phospholipid substrates play a role in which specific phospholipids are hydrolyzed by each enzyme type. We used PLA_2_ stereospecific inhibitors in tandem with a novel UPLC-MS/MS-based lipidomics platform to quantify more than a thousand unique phospholipid molecular species demonstrating cPLA_2_, sPLA_2_, and iPLA_2_ activity and specificity toward the phospholipids in living cells. The observed specificity follows the in vitro capability of the enzymes and can reflect the enrichment of certain phospholipid species in specific membrane locations where particular PLA_2_’s associate. For assaying, we target 20:4-PI for cPLA_2_, 22:6-PG for sPLA_2,_ and 18:2-PC for iPLA_2_. These new results provide great insight into the physiological role of PLA_2_ enzymes in cell membrane remodeling and could shed light on how PLA_2_ enzymes underpin inflammation and other lipid-related diseases.

The phospholipase A_2_ (PLA_2_) superfamily of enzymes contains six types that are designated based on their structural features (1). These include cytosolic PLA_2_ (cPLA_2_), secreted PLA_2_ (sPLA_2_), calcium independent PLA_2_ (iPLA_2_), and lipoprotein-associated PLA_2_, which is also known as platelet-activating factor acetyl hydrolase, as well as two less well studied types that were originally named for their source (lysosomal PLA_2_ and adipose associated PLA_2_). Subsequently, additional information about the already described PLA_2_’s and new PLA_2_’s has been reported and other nomenclatures referencing PLA_2_ activity have been independently developed, including acidic calcium-independent PLA_2_, alpha beta hydrolase domains, PLA/acyltransferases, glycosylphosphatidylinositol-specific PLA_2_’s, and patatin-like PLA_2_’s, some of which display PLA_2_ activity and are now considered to be part of the expanded PLA_2_ superfamily (2, 3). Note that some members of these later families have strong sequence homologies, but do not actually express PLA_2_ activity.

Each PLA_2_ type can consist of multiple groups and subgroups. For example, the cytosolic Group IV (GIV) cPLA_2_ group is comprised of at least six isoforms (A-F or α–ζ) in humans (4), while there are at least eleven mammalian sPLA_2_ Groups (GI, GII, GIII, GV, GIX, GX, GXII, GXIII, and GXIV), some of which are further divided into subgroups (5, 6) and at least nine Group VI (GVI) iPLA_2_s (A-I or β- ζ) (7, 8) have been described, each varying in sequence, molecular weight, tissue expression, and subcellular localization. PLA_2_ enzymes hydrolyze and release the acyl chain of membrane glycerophospholipids at the *sn-2* position, producing a lysophospholipid and free fatty acid (FA), both of which have important downstream proinflammatory signaling functions (9). This study focuses specifically on GIVA cPLA_2_, GV sPLA_2_, and GVIA iPLA_2_, which will sometimes be referred to herein for simplicity as cPLA_2_, sPLA_2,_ and iPLA_2_, respectively, and described findings may not be representative of all the specific members of the enzyme type and/or group/subgroup.

Considerable progress has been made toward describing PLA_2_ enzyme specificity toward the fatty acyl chain or polar headgroup components of their substrate glycerophospholipids. Previously, in vitro lipidomics studies with recombinant human PLA_2_ enzymes demonstrated striking differential specificities toward the glycerophospholipid acyl chain at the *sn*-2 position (10). Specifically, these studies showed cPLA_2_ has specificity toward arachidonic acid (AA, 20:4). On the other hand, it was discovered that the GV sPLA_2_ and GVIA iPLA_2_ preference was found to be toward linoleic acid (18:2) as well as myristic acid (14:0) (10). Acyl chain specificity was subsequently studied more closely by comparing ω-3 and ω-6 FAs at their *sn-2* position, indicating that ω-6 20:4 is the best substrate for GIVA cPLA_2_ and the ω-3 docosahexaenoic acid (DHA, 22:6) is the best substrate among these PUFAs for GV sPLA_2_ (11). Most importantly, we could explain the observed lipidomics-based specificities for each of the enzymes by detailed molecular dynamics simulations of the association of each enzyme with membranes and of the phospholipid substrate’s specific association and binding in the enzyme active site (10, 11).

While it has been suggested that the PLA_2_ enzyme specificity does not have a strong dependence on the chain length of the saturated FA at the *sn-1* position (10), PLA_2_ enzymes can distinguish well between *sn-1* alkyl ether or vinyl ether phospholipids (plasmalogens) (12). The in vitro polar head group dependence studies were predominantly carried out with four main types of phospholipids, two of which were zwitterionic and two of which were negatively charged, namely phosphatidylcholine (PC), phosphatidylethanolamine (PE), phosphatidylserine (PS), and phosphatidylglycerol (PG). It revealed that the cPLA_2_ did not show strong specificity toward any of the polar groups in equimolar mixtures of the phospholipids in Triton X-100/phospholipid mixed micelles, yet sPLA_2_ was shown to have some headgroup preference toward PG and iPLA_2_ toward PC in equimolar mixtures of phospholipids containing several different polar groups (10). Lastly, a recent study included the phosphatidylinositol (PI) headgroup and revealed a preference for cPLA_2_, but not for sPLA_2_ or iPLA_2_ (D. Hayashi, E. Dennis, 2024, manuscript in preparation). Despite progress in the field, our understanding of PLA_2_ enzymes specificity is fragmented and analytical methods to comprehensively study PLA_2_ enzymes ex vivo are lacking. In the current study, we present a novel method to simultaneously determine PLA_2_ preferences for both acyl chain and polar head group ex vivo using living RAW264.7 macrophage cells.

Macrophages are key mediators of inflammatory immune responses (13). Their effector functions are diverse and include the secretion of downstream PLA_2_ lysophospholipid products and free PUFAs which are the precursors of the proinflammatory eicosanoids and related bioactive lipids, sometimes referred to as oxylipins (14, 15). Our laboratory previously demonstrated a detailed comparative analysis of these signaling molecules across three types of primary mouse macrophages: resident peritoneal macrophages, thioglycolate-elicited macrophages, bone marrow-derived macrophages, and the immortalized macrophage-like RAW264.7 cell line (16). In the current study, RAW264.7 cells were chosen as a model system since they retain diverse macrophage effector functions, including expression of PLA_2_ enzymes, and can be cultured at scale for quantitative analysis of inflammatory metabolites such as various eicosanoids (17).

We previously established a targeted lipidomics approach to determine PLA_2_ substrate specificity in vitro. This approach utilized an HPLC/MS/MS system with a SCIEX 4000 Triple Quad MS using a hydrophilic interaction liquid chromatography column (10) to identify lysophospholipid products. We also developed a comprehensive system to analyze phospholipid molecular species from cells using a UPLC/MS/MS system with a SCIEX 6500 Triple Quad MS using normal, reversed phase and hydrophilic interaction liquid chromatography columns and compared the efficiency when a SelexION was included (18). We have now used a profiling approach with the use of a UPLC-MS/MS Q-Exactive Hybrid Quadrapole-Orbitrap exact mass spectrometer coupled with Lipid Data Analyzer (LDA) software especially designed for automatically identifying phospholipid molecular species among all of the complex lipids present (19, 20). This system offers the unique ability to separate, monitor, identify, and quantify over a thousand phospholipid molecular species from a single experiment.

Additionally, by using a polar end-capped C-18 reverse phase column, we are able to separate PS and other anionic phospholipids and lysophospholipids based on their FA chains as well as their polar head groups. These combined advantages allow for better species separation, fragmentation, and assignment, as determined using the “dynamic exclusion function” of MS/MS which allows for an increased number of fragmentations, and therefore increases coverage of species identification. Here, we report a comprehensive method that allows for identification of all major phospholipids including the specific acyl chains at the *sn-1* and *sn-2* position in living cells.

## Materials and Methods

### Cell culture

The RAW264.7 murine macrophage cell line (ATCC #TIB-71) was maintained at 37°C, 5% CO_2_ in DMEM containing 10% fetal bovine serum which includes albumin, 1% penicillin/streptomycin and L-glutamate (hereafter designated as “media”). Unless otherwise stated, cells were treated, then placed on ice, and washed three times with ice-cold Dulbecco’s PBS. One milliliter of ice-cold Dulbecco’s PBS was added to each well, and cells were scraped using rubber scrapers, transferred to 1.5 ml Eppendorf tubes, and centrifuged at 20,000*g* for 20 min. After discarding the supernatant, the cell pellet was resuspended in 200 μl of 10% methanol and used for extraction or stored at −80°C.

For supplementation with exogenous free FAs, cell viability assays were performed (data not included) showing the same survival rate for those exposed to media-containing FAs as those containing just media.

### cPLA_2_ and sPLA_2_ assay

**Fig. 1:**
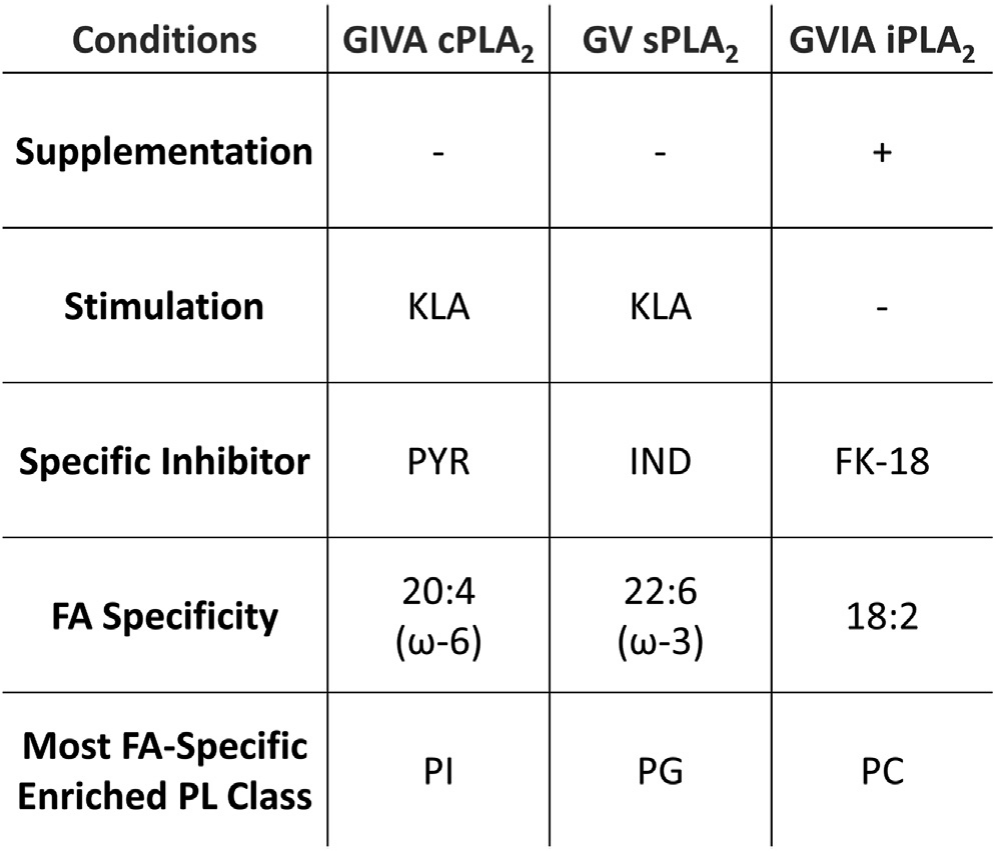
Summary of assays. Table showing differences between the specific assay conditions as well as the phospholipid molecular species used to determine the substrate specificity for c-, s- and iPLA_2_. cPLA_2_, cytosolic phospholipase A_2_; iPLA_2_, calcium independent phospholipase A_2_; sPLA_2_, secreted phospholipase A_2_.

Cells were plated in a 12-well plate in 1 ml DMEM at the concentration of 0.23 x 10^6^ cells per well and left overnight to adhere. Media were changed, and cells were treated with vehicle (Kdo_2_ lipid A, KLA) or an inhibitor, or both for 30 min: cPLA_2_ inhibitor, PYR, (21) (pyrrophenone, Cayman Chemical) at a concentration of 1 μM per well or sPLA_2_ inhibitor, IND, (22) (LY315920, Selleck Chemicals) at a concentration of 100 μM per well. Afterward, KLA was added (Avanti Polar Lipids) at a concentration of 100 ng/ml, and cells were incubated for 24 h. The time course for cPLA_2_ activity had been performed (data not shown) by measuring the levels of eicosanoids, produced in response to 20:4 release. We found that even though cPLA_2_ shows activation after, as little as 1 h of KLA treatment, the level of eicosanoids was significantly higher at 6 h and leveled off at 24 h, and we chose the latter for our assay so all the effect of all three enzymes on the phospholipid composition would be assayed at the same time point. The choice of inhibitors was based on the previous in vitro detailed kinetic study with these three enzymes (23). The differential assays are summarized in Fig. 1.

### iPLA_2_ assay

Cells were plated in a 12-well plate with 1 ml of media at a concentration of 0.1 x 10^6^ cells per well and were then left overnight to adhere. When free FAs were supplemented, they were first added to the media to first expose the cells to the FAs in the presence of albumin. Media were replenished unsupplemented or supplemented with 25 μM of linoleic acid (18:2), myristic acid (14:0), AA (20:4), or DHA (22:6) and incubated for 24 h. Afterward, media were replenished and again supplemented with either media or media supplemented with 25 μM of FA.

Kokotos *et al.* developed an iPLA_2_ specific class of fluoroketone inhibitors, of which FKGK18 was the most potent (24). Ramanadham et al., showed that this inhibitor works optimally in living cells (25). RAW264.7 cells were then treated with media or media supplemented with iPLA_2_ inhibitor–FK18 (FKGK 18, Cayman Chemical) at a concentration of 30 μM per well for 24 h. The assay is summarized in Fig. 1.

### Statistical analysis

Data were analyzed using the unpaired *t* test with Welch's correction unless otherwise stated. Statistical significance was marked with an asterisk: ∗*P* < 0.05, ∗∗*P* ≤ 0.01, ∗∗∗*P* ≤ 0.001, ∗∗∗∗*P* ≤ 0.0001, and ns = not significant. All experiments were performed in biological triplicates.

### Extraction of lipids

First, a precise aliquot of EquiSPLASH™ LIPIDOMIX® Quantitative Mass Spec Internal Standard (Avanti) containing deuterated phospholipids (an equimolar mixture of PC, Lyso PC, PE, Lyso PE, PI, PS, and PG) was spiked into 500 μl of 264.7 RAW cells homogenized in 10% methanol in water. Aliquots containing 1.8 mg of protein (based on the Quick Start Bradford assay measuring A550) were used to validate the methodology. Lipids were extracted using a modified BUME method (26). A solution of 500 μl of 3:1 butanol/methanol was added, vortexed, and sonicated for 10 min. Solutions of 500 μl of 3:1 heptane/ethyl acetate and 500 μl 1% acetic acid were added. Samples were vortexed to form an emulsion and centrifuged at 5,000 rpm for 5 min to form two distinct layers. The top layer containing the lipid was transferred to a max recovery MS vial (Phenomenex) to minimize the dead volume for LC-MS injection. The extracts were brought to dryness using a speed vac and reconstituted in 50 μl of 18:1:1 isopropanol/dichloromethane/methanol. To ensure that the method could identify a variety of phospholipid molecular species not usually seen in RAW cells, the same extraction conditions were used on 20 μl of human plasma obtained from the United States National Institute of Standards and Technology and bovine brain extract (1 mg/ml, Avanti).

### Reverse-phase chromatography

Lipids were analyzed on a Vanquish UHPLC (Thermo Fisher Scientific) mass spectrometer using a T3 1.6 μM 2.1 mm × 150 mm column (Waters). This is a polar end-capped C18 column that works on the principle of reverse-phase chromatography with some polar resolution. Under conditions of reverse phase chromatography, several lipids, including PS tend to elute as broad peaks. To address this issue, we have developed an elution method that in combination with the T3 column improves the resolution and prevents any peak trailing. To achieve optimal resolution of the lipid molecular species based on the FA composition, we used two methods: shorter (30 min) and longer (60 min).

#### The 30-minute method

This method consists of a step gradient from 25% buffer A (10 mM ammonium formate and 1% formic acid in water) to 100% buffer B (70/30 isopropanol/acetonitrile with 10 mM ammonium formate and 1% formic acid) over 35 min. The gradient starts at 25% B from 0 to 0.5 min, ramps to 60% B at 2 min, ramps to 75% B at 7 min and holds until 15 min, ramps to 80% B at 22 min, ramps to 95% B at 26 min, ramps to 100% B at 33 min and holds until 35 min. Flow rate was set at 0.3 ml/min.

#### The 60-minute method

To achieve optimal resolution of the FA isomeric species, we used a step gradient from 25% buffer A (10 mM ammonium formate and 1% formic acid in water) to 100% buffer B (70/30 isopropanol/acetonitrile with 10 mM ammonium formate and 1% formic acid) over 60 min. The gradient starts at 25% B from 0 to 1 min, ramps to 60% B at 4 min, ramps to 70% at 14 min, ramps to 75% B at 40 min, ramps to 99% B at 57 min, then holds until 59 min. Gradient ramps down to 25% B at 60 min. Flow rate was set at 0.3 ml/min.

### MS/MS analysis

The chromatography column was interfaced with a Q-Exactive Hybrid Quadrapole-Orbitrap mass spectrometer (Thermo Fisher Scientific). Ion source parameters were as follows: sheath gas 48 AU; aux gas 11 AU; sweet gas 1 AU; and spray voltage 3.5 kV for positive mode and 2.5 kV for negative mode; capillary temperature 250°C; S-lens RF level 60 AU; and aux gas heater temperature 413°C. Lipid species were analyzed using a data-dependent acquisition Top N scan of 8 with a nominal collision energy of −30 in negative mode and an nominal collision energy of +25 in positive mode. Ions between 200-1,200 m/z were monitored. MS1 resolution was set at 70,000 (FWHM at m/z 200) with an automatic gain control target of 1e6 and maximum IT of 200 ms. MS2 resolution was set to 17,500 with an automatic gain control target of 5e4, fixed first mass of 80 m/z, and maximum IT of 50 ms. The isolation width was set at 1.2 m/z, and the dynamic exclusion was set at 3 s. If Idle, pick others was selected. Lipid identification and quantification was carried out with LDA software, version 2.8.1 (19, 20), which included phospholipids, sphingolipids, and glycerolipids and is now further extended to include cholesterol esters, acyl carnitines, and plasmalogens (see Lipid Data Analyzer (LDA) additions in Supplemental data for details).

**Fig. 2:**
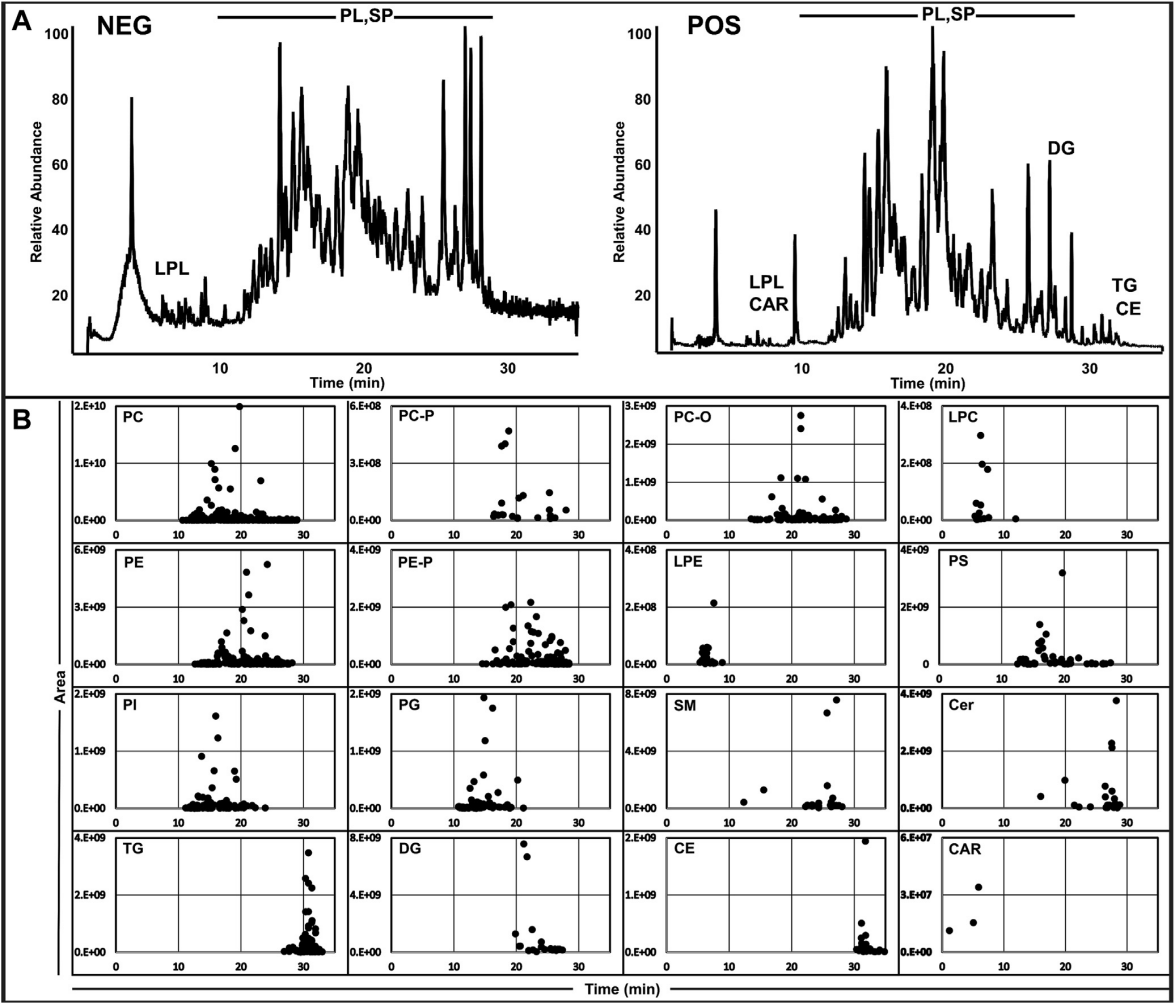
RAW 264.7 cells. A: Chromatogram of 100 μl of RAW 264.7 cells (1.8 mg protein) homogenized into 500 μl 10% methanol in negative and positive mode using 30 min gradients. B: Abundance and elution time of lipid molecular species confirmed by fragmentation pattern and separated by lipid class. Each point represents one major molecular species.

Reverse-phase chromatography coupled with MS/MS analysis allow a comprehensive analysis of phospholipids, glycerolipids, sphingolipids, cholesterol esters, and acylcarnitines from a broad range of biological samples which include, but are not limited to, macrophage RAW264.7 cells (Fig. 2), National Institute of Standards and Technology plasma (Supplemental Fig. S1) and brain tissue, which is rich in anionic phospholipid species (Supplemental Fig. S2). Some of the phospholipid polar classes can be found in both positive and negative mode. For example, PC species can be found in positive mode using the [M+H]^+^ ion and in the negative mode using the [M+HCOO]^−^ ion or the [M-CH_3_] ^−^ ion.

We also explored two separation methods, one using a steeper (30 min) and one with a shallower (60 min) gradient for optimal resolution of phospholipid species (Supplemental Fig. S3). We ultimately used the shorter one for the data presented in this paper. The results presented in this manuscript consist of phospholipid data from the negative mode, though the sphingolipids, glycerol lipids, sterol lipids, and acyl carnitines are also simultaneously identified when run in the positive mode. Note that some groups, such as PI, do not form a positive ion and therefore cannot be detected in positive mode. Another advantage of the negative mode is that we can identify the lipid molecular species by carboxy fragments only formed in negative mode (see Supplemental data). The ionization of each molecular species depends on its FA composition, and only one deuterated internal standard was used for each phospholipid class (see Lipid Extraction above). The data have been normalized to the internal standard of its phospholipid class and is shown in arbitrary units (A.U.) relative to that internal standard.

## Results

### Phospholipid PUFA composition in RAW cells by polar group

**Fig. 3:**
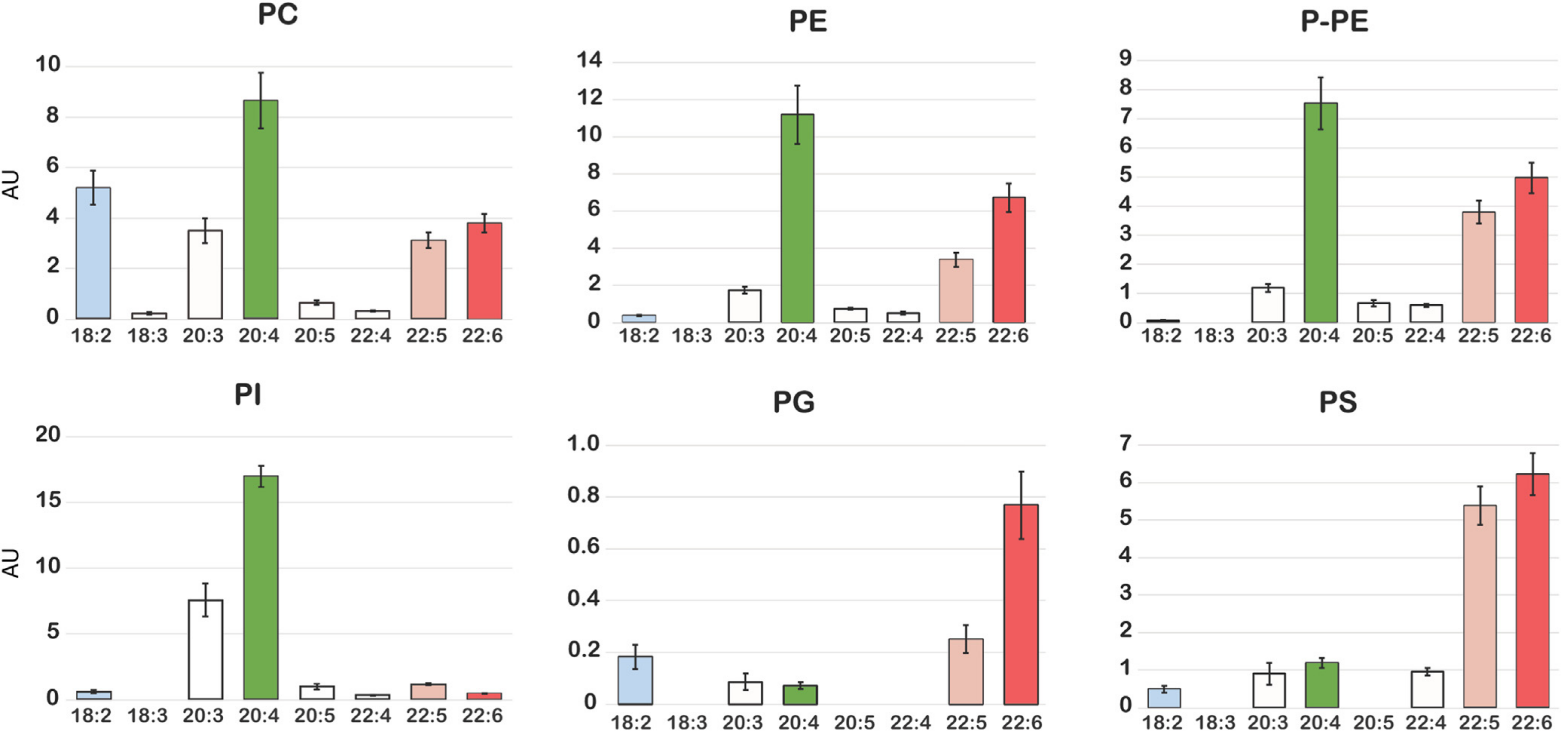
Polar group phospholipid PUFA composition in RAW cells. PC has significant levels of 18:2 (blue) as well as 20:4 (green) and 22:5/22:6 (pink and red, respectively). PE and P-PE show similar profiles to PC but lack much 18:2. PI is enriched in 20:4, while PG and PS is most enriched in 22:5/22:6. PC, phosphatidylcholine; PE, phosphatidylcholine; PG, phosphatidylglycerol; PI, phosphatidylinositol; P-PE, plasmalogen of phosphatidylethanolamine; PS, phosphatidylserine.

For the majority of the phospholipids, LDA was able to distinguish between the *sn-1* and *sn-2* positions. Saturated FAs occupied 73% of the *sn*-1 position, while unsaturated FAs occupied 27% of the *sn*-1 position but were primarily 18:1. For the *sn*-2 position, unsaturated FAs occupied 94% of the *sn*-2 position, most of which were PUFAs with only 18% 18:1 (data not shown). Therefore, for simplicity, we did not include phospholipids that contained two saturated (16:0, 18:0) or monounsaturated (18:1) FAs in this analysis. Conveniently, the phospholipid polar classes of RAW cells exhibit an optimal distribution of PUFAs, aligning with previous findings indicating a preference for various PLA_2_ enzymes in in vitro specificity assays. Specifically, for cPLA_2_ 20:4 is enriched at the *sn-2* position in PC, PE, plasmalogen of phosphatidylethanolamine (P-PE), and PI. For sPLA_2_, 22:6 is enriched at the *sn-2* position in PC, PE, P-PE, PG, and PS. Finally, for iPLA_2_, 22:6 is almost exclusively in PC. Moreover, 22:5, the immediate precursor of 22:6 biosynthetically follows closely the distribution of 22:6. Interestingly, PI is highly enriched in 20:4 among the polar groups, while PG is highly enriched in 22:6 among the polar groups and 18:2 is highly enriched in PC (Fig. 3). For initial studies of PLA_2_ specificity in cells, we focused on these three most abundant phospholipid molecular species PI/20:4, PG/22:6, and PC/18:2 to reflect the in vitro predictions.

### Analysis of PUFA release—KLA stimulation

**Fig. 4:**
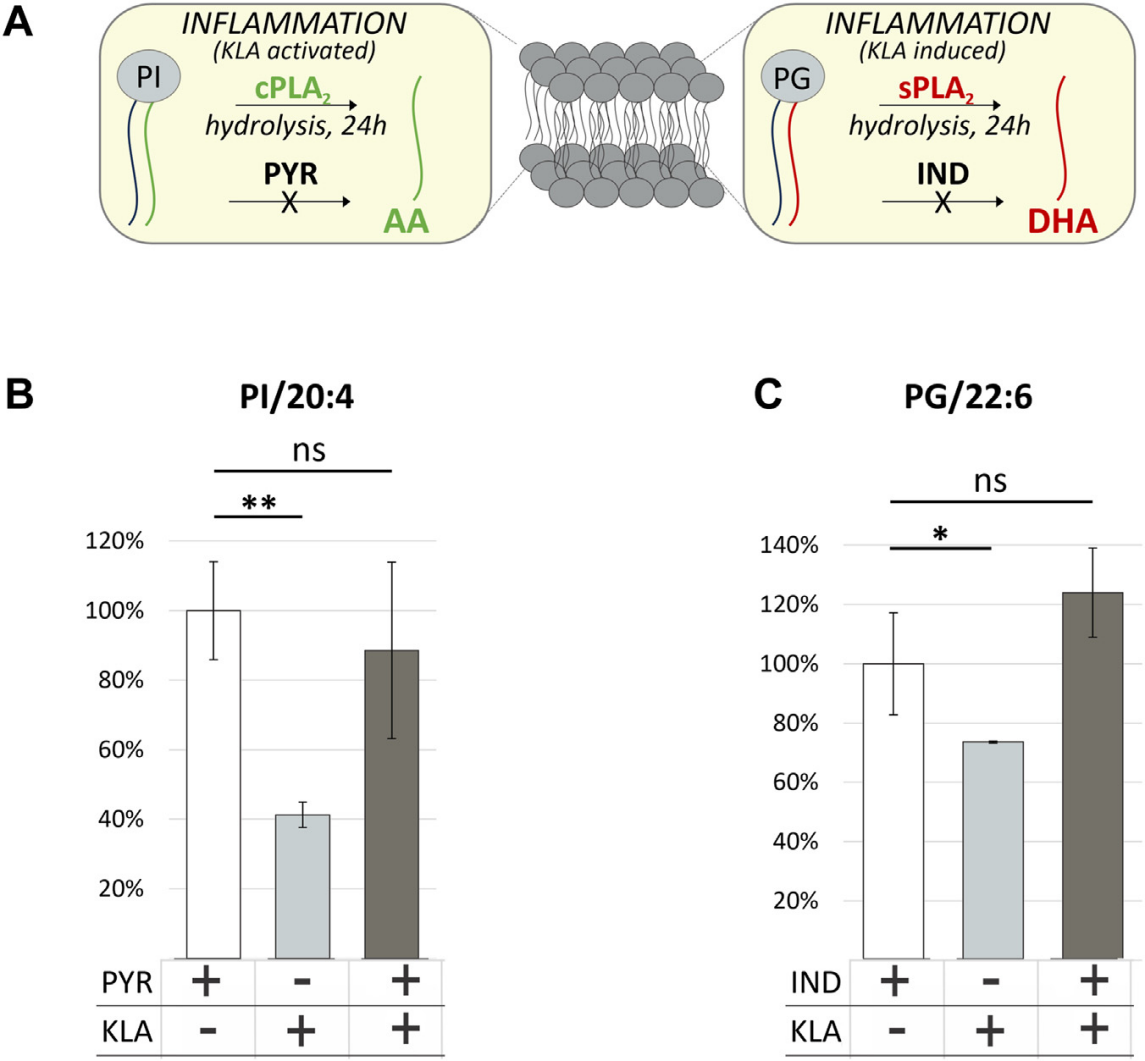
cPLA_2_ and sPLA_2_ assays. A: Specific conditions for assay of GIVA cPLA_2_ and GV sPLA_2_. B: cPLA_2_ activity toward *sn-2* 20:4 PI. Control (white), 24 h KLA stimulation (light gray), cPLA_2_ inhibitor (PYR) pretreatment followed by 24 h KLA stimulation (*dark gray*); C: sPLA_2_ activity toward sn-2 22:6 PG. Control (white), 24 h KLA stimulation (*light gray*), sPLA_2_ inhibitor (IND) pretreatment followed by 24 h KLA stimulation (*dark gray*). AA, arachidonic acid (20:4), DHA, docosahexaenoic acid (22:6). cPLA_2_, cytosolic PLA_2_; KLA, Kdo_2_ lipid A; PG, phosphatidylglycerol; PI, phosphatidylinositol; PLA_2_, phospholipase A_2_; sPLA_2_, secreted PLA_2_.

When stimulated with the toll-like receptor-4 (TLR-4) activator lipopolysaccharide or a chemically defined version KLA, cPLA_2_ (27) is activated and sPLA_2_ (28) expression is induced in RAW264.7 cells. cPLA_2_ and sPLA_2_ activity toward PI/20:4 and PG/22:6 phospholipids is schematically shown in Fig. 4A, and was measured in the presence or absence of their respective inhibitors upon cotreatment with KLA. A large effect was observed for PI/20:4, showing 50% reduction in 20:4 when stimulated with KLA that could be rescued following cotreatment with the cPLA_2_ specific inhibitor, PYR, indicating a clear cPLA_2_ activity preference toward 20:4 in PI (Fig. 4B and Supplemental Fig. S4). Similar reductions were observed for PG/22:6 following treatment with KLA. Levels of 22:6 were restored upon cotreatment with the sPLA_2_ specific inhibitor, IND, indicating an sPLA_2_ activity preference for 22:6 (Fig. 4C and Supplemental Fig. S5). To validate the assay, the experiment was repeated with additional PLA_2_ specific inhibitors. Indeed, PI/20:4 hydrolysis was only blocked with PYR (Supplemental Fig. S6A), while PG/22:6 hydrolysis was only blocked by IND (Supplemental Fig. S6B). These findings are consistent with the reported specificities of PYR (21) and IND (22) and collectively validate our analytical approach to study cPLA_2_ and sPLA_2_ activity in living cells.

### Effects of PUFA supplementation on membrane composition

Upon cell supplementation with each respective FA, absolute levels were observed to increase compared to nonsupplemented conditions, yet the overall distributions remain generally unaltered: PC enriched in 18:2, PI enriched in 20:4, and PG enriched in 22:6 (Supplemental Fig. S7). Notably, supplementation with 18:2 results in increased levels of 18:2 in PC, as well as its elongated product, 20:4. Conversely, supplementation with 20:4 does not significantly elevate the levels of 20:4 in PI. Supplementation with 22:6 shows a striking increase in 22:6 levels across all major polar groups, including PG.

### Analysis of PUFA release after supplementation—iPLA_2_ activity

**Fig. 5:**
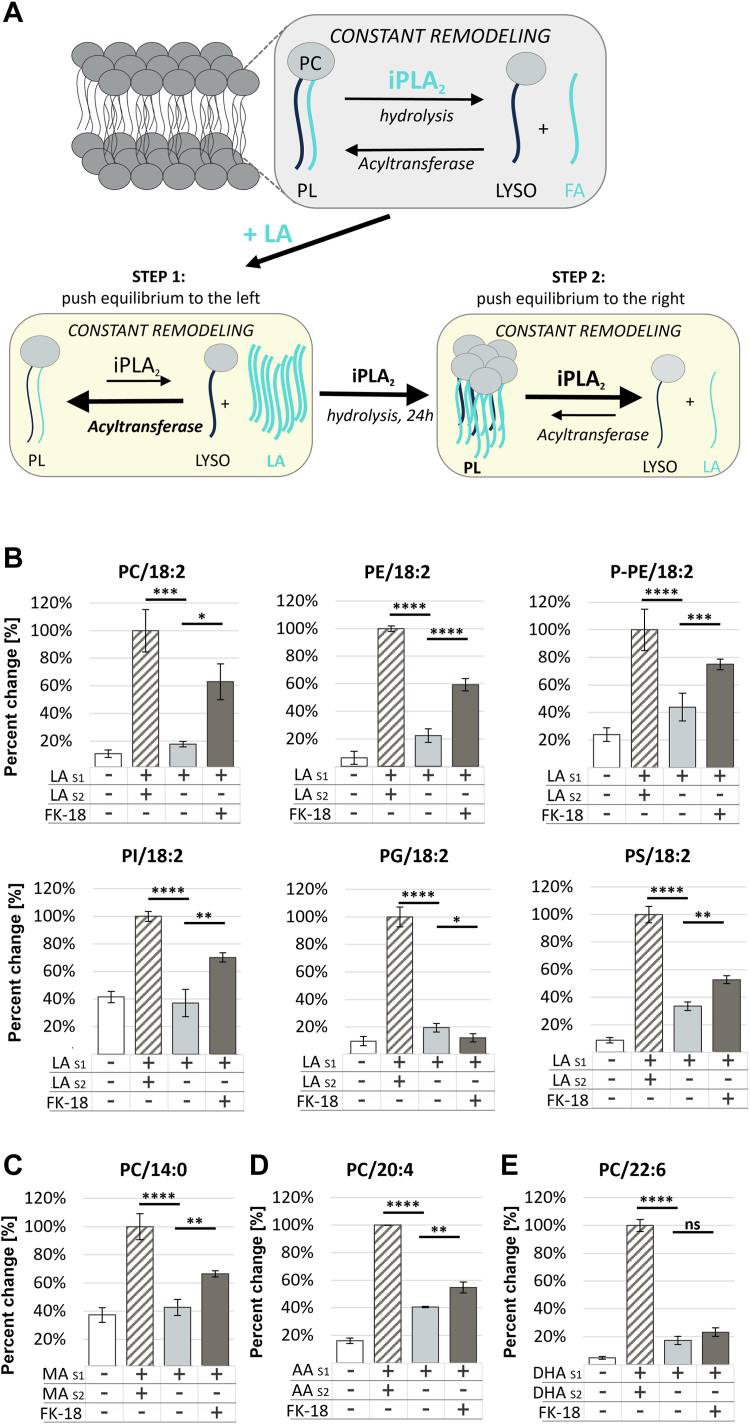
iPLA_2_ assay (A) Specific conditions for assay of GVIA iPLA_2_. B: iPLA_2_ activity toward *sn-2* 18:2 PC, PE, P-PE, PI, PG, and PS in cells supplemented with 18:2. C: iPLA_2_ activity toward sn-2 14:0 PC in cells supplemented with 20:4. D: iPLA_2_ activity toward sn-2 20:4 PC. E: iPLA_2_ activity toward sn-2 22:6 PC in cells supplemented with 22:6. Negative control (*white*), nonsupplemented cells, positive control (*stripes*), cells supplemented with B. 18:2 FA (or C. 14:0, D. 20:4, or E. 22:6 where noted) for 48 h, iPLA_2_ activity measured for 24 h (*light gray*), iPLA_2_ inhibitor (FK-18) for 24 h (*dark gray*). LA_S1_ and LA_S2_, first and second supplementation with FA 18:2, 14:0, 20:4, or 22:6 for 24 h. iPLA_2_, calcium independent phospholipase A_2_; PC, phosphatidylcholine; PE, phosphatidylcholine; PG, phosphatidylglycerol; PI, phosphatidylinositol; PLA_2_, phospholipase A_2_; P-PE, plasmalogen of phosphatidylethanolamine; PS, phosphatidylserine.

Unlike cPLA_2_ and sPLA_2_, iPLA_2_ lacks an activating "ON" switch such as KLA. To evaluate iPLA_2_ activity, we developed a method to skew the steady state equilibrium phospholipid profile. This was achieved by supplementing cells with FA to maximize its incorporation into phospholipids. Subsequently, the supplemented media were replaced with unsupplemented media, and iPLA_2_ activity was monitored by the change in glycerophospholipid content (Fig. 5A).

Previous in vitro studies as well as molecular dynamic simulations showed that iPLA_2_ has two hydrophobic binding pockets: one containing aromatic residues that accommodates the linoleic acid tail and the other containing exclusively aliphatic residues and accommodates the myristic acid tail (10). Therefore, to investigate iPLA_2_ activity toward linoleic acid, we supplemented cells with 18:2 and measured iPLA_2_ activity upon removing the supplement from the media (Fig. 5B). The data show release of 18:2 from PC, but also from the other major polar groups, which was blocked in the presence of an iPLA_2_ specific inhibitor FK-18 (Fig. 5B) but not cPLA_2_ or sPLA_2_ specific inhibitors, indicating that neither of those enzymes influence the reaction (Supplemental Fig. S8). These data demonstrate that iPLA_2_ does not show strong preference toward any particular polar group, yet it presents a strong specificity toward 18:2. However, as shown in Fig. 3, the majority of the 18:2 accumulates in PC; therefore, we used PC/18:2 to assay iPLA_2_.

To ensure that iPLA_2_ does not compete with cPLA_2_ or sPLA_2_, the cells were supplemented with 18:2, ensuring high content of linoleic acid across all phospholipid headgroups, and stimulating with KLA to activate cPLA_2_ and sPLA_2_. Increases in 18:2 levels were observed in the presence of KLA regardless of polar headgroup identity, indicating that neither cPLA_2_ nor sPLA_2_ was acting significantly on 18:2, probably because of the low levels of 18:2 in PI or PG compared with 20:4 and 22:6, respectively, thus demonstrating the lack of substrate competition (Supplemental Fig. S9).

We next explored the second hydrophobic binding pocket of iPLA_2_ with an assay based on myristic acid (14:0). Indeed, the enzyme showed activity toward 14:0, but only in PC and PE indicating somewhat reduced activity compared to 18:2 (Fig. 5C, Supplemental Fig. S10B).

### Analysis of PUFA release after supplementation—exploring other substrates for iPLA_2_

Molecular dynamics indicated an 18:2 elongated product, 20:4 was poorly accommodated in a combined pocket of iPLA_2_ in an unstable binding mode (10). In accordance with these predictions, we detected the enzyme activity only toward PC/20:4 (Fig. 5D, Supplemental Fig. S10C). While the activity of iPLA_2_ can be measured, the levels of PC/20:4 are still twice as high as before supplementation and the recovery with FK-18 inhibitor are minimal. These data experimentally support the molecular dynamics prediction that 20:4 is not a preferred substate for iPLA_2_.

Similar molecular dynamics predictions exclude PC/22:6 as a substrate for iPLA_2_. However, here, we report that 22:6 is preferred more than 20:4, but less than 18:2. Specifically, iPLA_2_ hydrolyzes the PE/22:6, PS/22:6, and PG/22:6 (Fig. 5E, Supplemental Fig. S10D), possibly due to a larger number of π- π interactions between the 22:6 double bonds (which are ω −3 vs. 20:4 which is ω −6) and the aromatic-rich binding site of iPLA_2_ (10).

To address previous in vitro findings that eicosapentaenoic acid (20:5) could serve as a good substrate for iPLA_2_ (11), we supplemented the cells with 20:5 but observed that a majority becomes quickly elongated to 22:5 and 22:6 species across all major phospholipid subclasses (Supplemental Fig. S11), which makes it challenging to use it as a supplement for these assays.

In summary, iPLA_2_ being constitutively active, can act on other FAs, such as 20:4 and 22:6, yet its preferred substrate is 18:2. This can be explained by previous reports indicating that iPLA_2_ localizes and acts on mitochondrial phospholipids (29, 30), which are very high in 36:2 acyl chains in PC and PE (31). We hypothesize that the acyl chains reported as 36:2 are likely *sn-1* 18:0 and *sn-2* 18:2, making it a perfect substrate for iPLA_2_.

## Discussion

**Fig. 6:**
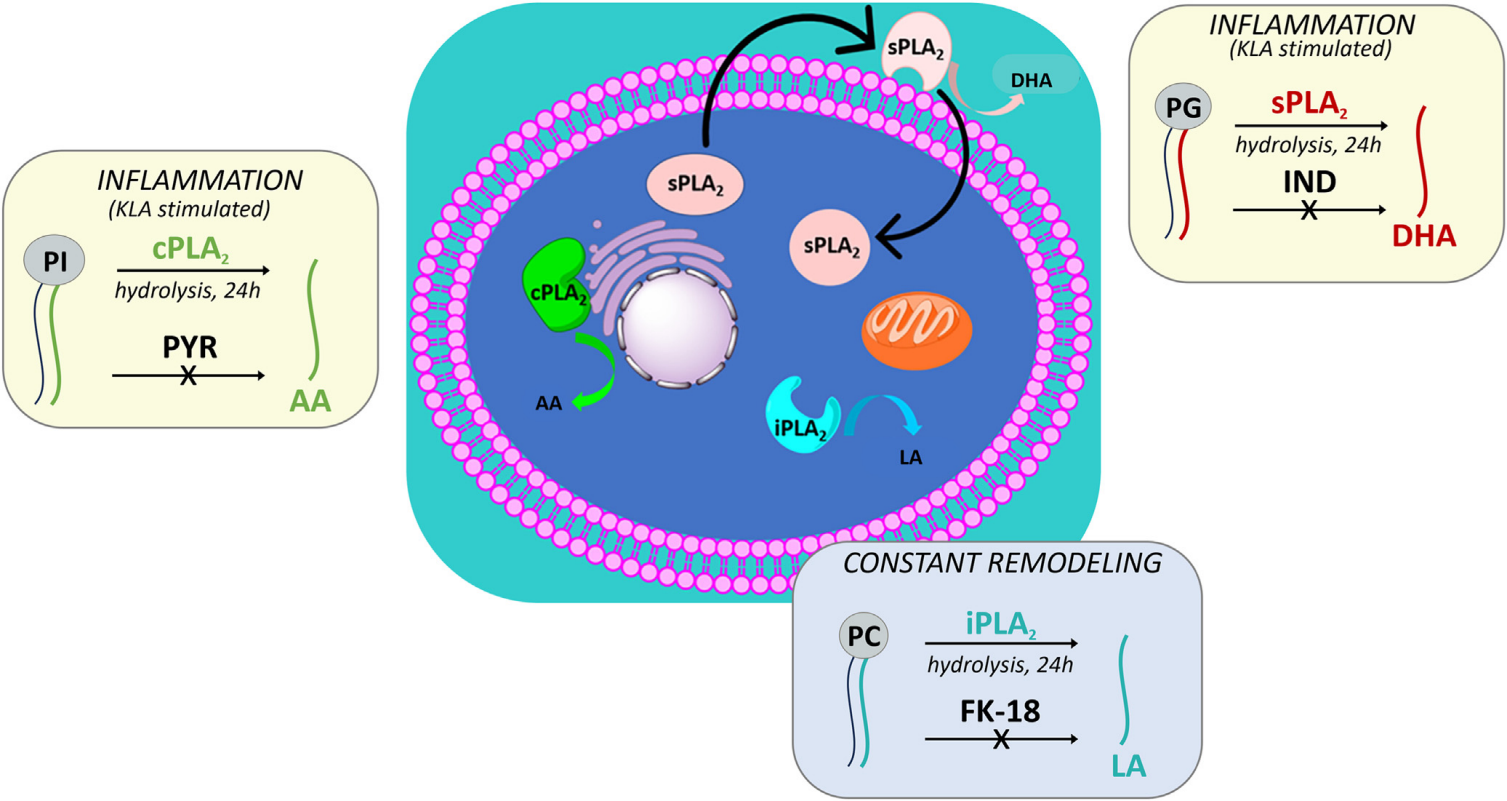
Summary of phospholipase A_2_ specificity and function in macrophages. Cartoon representation of GIVA cPLA_2_, GVIA iPLA_2_, and GV sPLA_2_ depicting the main product of their hydrolysis as well as their reported cellular localizations. cPLA_2_ translocates to the perinuclear membranes and is found to be active on arachidonic acid (AA, 20:4). iPLA_2_ is found in the cytosol and associated with mitochondria, where it hydrolases linoleic acid (LA, 18:2) from phospholipids. sPLA_2_ is found to be secreted and acts on extracellular phospholipids releasing mainly docosahexaenoic acid (DHA, 22:6). cPLA_2_, cytosolic PLA_2_; iPLA_2_, calcium independent phospholipase A_2_; sPLA_2_, secreted PLA_2_.

We have developed a comprehensive UPLC-MS/MS method to separate, assign, and identify thousands of phospholipid molecular species in RAW macrophage cells as well as in human plasma and brain tissue rich in anionic phospholipids. We used this methodology to showcase three major PLA_2_ enzymes and their substrate specificity in living cells. We report cPLA_2_ specificity toward AA 20:4 from PI, while sPLA_2_ releases mainly DHA from PG. Additionally, we developed an assay to study iPLA_2_ membrane remodeling activity, where it shows preference toward linoleic acid (18:2) from almost all major phospholipid headgroups with minimal substrate competition from the other PLA_2_’s (Fig. 6). Our phospholipid analysis shows that most of the phospholipids contain a PUFA at the *sn*-2 position across the six main polar groups. Therefore, our analysis focused on changes in PUFAs for each enzyme.

It is important to mention that 20:4, which is the major PUFA in the membranes of innate immune cells, is not uniformly distributed among membrane glycerophospholipids. As reported here in the RAW 264.7 cell line, PI is the richest 20:4 containing class, followed by PE, P-PE, and PC. In the resident peritoneal macrophages and human monocytes, that distribution may be different in detail (32, 33). A recent study reports cPLA2γ which differs from the KLA activatable cPLA_2_α was shown to be involved in constitutive 20:4 phospholipid remodeling between PC and PE by direct transacetylation (34). It shows the importance of retaining 20:4 in the appropriate phospholipid pools (32, 33) and contributions to retaining the asymmetric distribution of 20:4 between phospholipid headgroups. Studies suggest that some enzymes release 20:4 from PC, but not PE or PI (35), indicating that the phospholipid molecular species availability at a given enzyme’s subcellular location might be the rate limiting step to its observed activity in cellular systems. This would support our hypothesis that the PLA_2_^’^s substrate specificity expressed *ex vivo* is limited by their subcellular location, and hence the availability of the optimal FA on the optimal polar headgroup in the membrane phospholipids.

We wish to point out that we have deliberately focused on the specific activity of three main PLA_2_’s in the murine RAW 264.7 macrophage cell line for these studies as a prototype for determining the ex vivo specificity of these enzymes in living cells. We are fully aware that specific PLA_2_ groups and subgroups and their expression levels differ between primary macrophages and transformed cell lines (16) as well as between different cell types and tissues. Clearly similar enzymes in other species may have different specificity, yet the in vitro specificity of these three enzymes when expressed recombinantly with human sequences demonstrate overall similar specificity. However, this study is specifically focused on murine GIVA cPLA_2_, GV sPLA_2_, and GVIA iPLA_2_. The RAW cells may express other PLA_2_s which could contribute to the observed activity ex vivo. Other PLA_2_ types (such as lipoprotein-associated PLA_2_ and acidic calcium-independent PLA_2_) have been shown to act on oxidized FAs in phospholipids (36, 37, 38, 39, 40), which is beyond the scope of these studies. In the future genetic approaches such as siRNA KO and CRISPR-Cas9 knockdown data could add support to our conclusions.

## Data availability

Data will be shared upon written request to Edward Dennis at UCSD through email edennis@ucsd.edu. LDA software is available for download and individual use at: http://genome.tugraz.at/lda2.

## Supplemental data

This article contains supplemental data.

## Conflict of interest

The authors declare that they have no conflicts of interest with the contents of this article.
