# Supplementary material for: Lipidomics of phospholipase A2 reveals exquisite specificity in macrophages
Source: J Lipid Res. 2024 May 23;65(7):100571. doi: 10.1016/j.jlr.2024.100571 (PMC11254598; doi:10.1016/j.jlr.2024.100571)
Supplement: Supplemental Data [file mmc1.docx]

**SUPPLEMENTAL DATA**

**Lipidomics of Phospholipase A_2_ Reveals Exquisite Specificity in Macrophages**

Gosia M. Murawska, Aaron M. Armando, Edward A. Dennis^*^

Department of Chemistry and Biochemistry and Department of Pharmacology, School of Medicine, University of California at San Diego, La Jolla, CA 92093, USA

**^*^Corresponding author**: Edward A. Dennis (edennis@ucsd.edu)

**ADDITIONAL METHODS**

**Lipid Data Analyzer (LDA) Additions**

*Alkyl Ether and Vinyl Ether Phosphatidylcholine*

Alkyl ether (PC-O) and vinyl ether (PC-P) phosphatidylcholines ionize in negative mode as [M+HCOO]^-^ and [M-CH_3_]^-^. Using this method, we found the former ion to be more abundant and we use this for identification. PC species can be identified by a neutral loss of formate and CH_3_ or C_2_O_2_H_4_ (*m/z* 60.02), C_4_H_11_NO_4_P (m/z 168.04), and C_7_H_15_O_5_NP (m/z 224.07). Chain identification and positions are determined by fragments corresponding to the ring fragment (neutral loss of PC head 60, carboxy, and C_4_H_9_N), carboxy, alkenyl, neutral loss of the carboxy, and neutral loss of the ketene. PC-O species do not fragment at the PC head 224 or alkenyl locations and can be separated from PC-P species by LDA using these distinctions. There is also a retention time difference that can separate PC-O and PC-P isobars as the double bond is in the middle of the fatty acid in PC-O and adjacent to the ether as a vinyl ether in PC-P. This double bond difference causes the PC-P species to elute later in reverse phase.

*Phosphatidylethanolamine Plasmalogen (P-PE)*

LDA Software identified the P-PE molecular species in the negative mode (18).  The PE headgroup is identified by two fragments of [C_2_H_7_O_4_NP]^-^ at m/z 196.036 and [C_5_H_11_O_5_NP]^-^ at m/z 140.011. The fragments used for the *sn-2* chain identification are the carboxy group, neutral loss of the ketene, and neutral loss of the carboxy. The fragment used to identify the *sn-1* chain contains the alkenyl.  For example, PE P-18:0/22:6 would give a fragmentation pattern of: Precursor [C_45_H_77_O_7_NP]^-^ at m/z 774.543, neutral loss of ketene at [C_23_H_47_O_6_NP]^-^ 464.318, neutral loss of carboxy [C_23_H_45_O_5_NP]^-^ at m/z 446.309, carboxy group 22:6 [C_22_H_31_O_2_]^-^ at m/z 327.236, alkenyl P-18:0 [C_18_H_35_O]^-^ at m/z 267.269, and PE head fragments of [C_5_H_11_O_5_NP]^-^ at m/z 196.036 and [C_2_H_7_O_4_NP]^-^ at m/z 140.011.  Due to the lack of available standards for phospholipid species containing alkyl ether PE (O-PE), we do not try to detect any PE molecular species with alkyl ethers at the *sn-1* position, unless present in significant quantity. Noise filtering and fragmentation intensity rules were established to prevent false positive identification.

*Cholesterol Esters*

Cholesterol esters ionize in positive mode as [M+NH_4_]^+^. The fatty acid fragments with the ammonia as a neutral loss leaving the cholesterol fragment of C_27_H_45_ (*m/z* 369.35). LDA uses the exact mass and the cholesterol fragment to identify each cholesterol ester species.

*Acyl Carnitines*

Acyl Carnitines ionize in positive mode as [M+H] ^+^. The carnitine loses the C_3_H_9_N (*m/z* 59.07) and acyl chain as neutral fragments leaving the C_4_O_2_H_5_ (*m/z* 85.03). LDA uses the exact mass of carnitine, and the neutral loss of carnitine (Precursor-C_3_H_9_N) ions to identify acyl carnitine species.

**FIGURES**

**
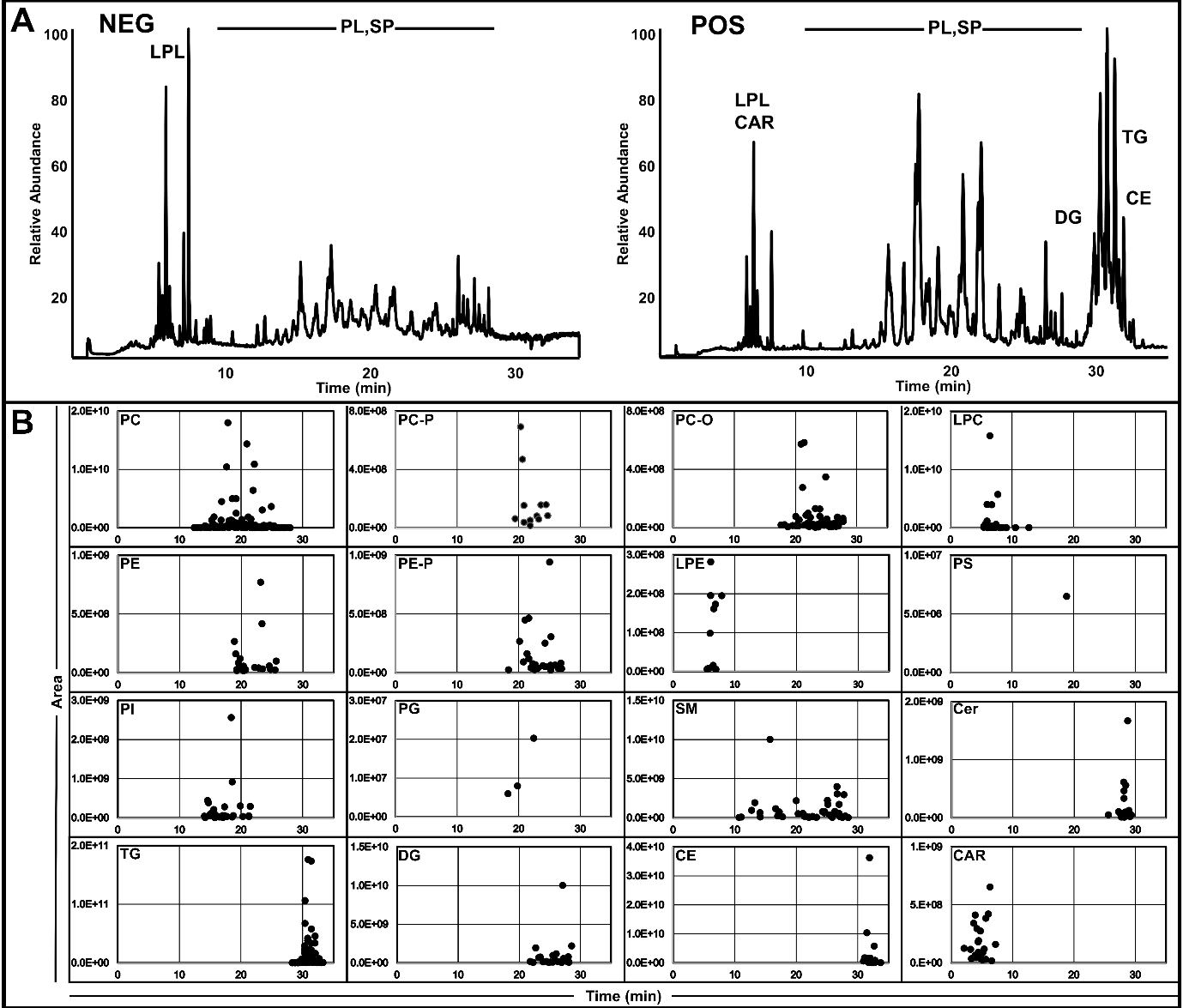
**

S 1. NIST Plasma. A: Chromatogram of 20 µL NIST human plasma in negative and positive mode using 30 min gradients. B: Abundance and elution time of lipid molecular species confirmed by fragmentation pattern and separated by lipid class. Each point represents one major species.


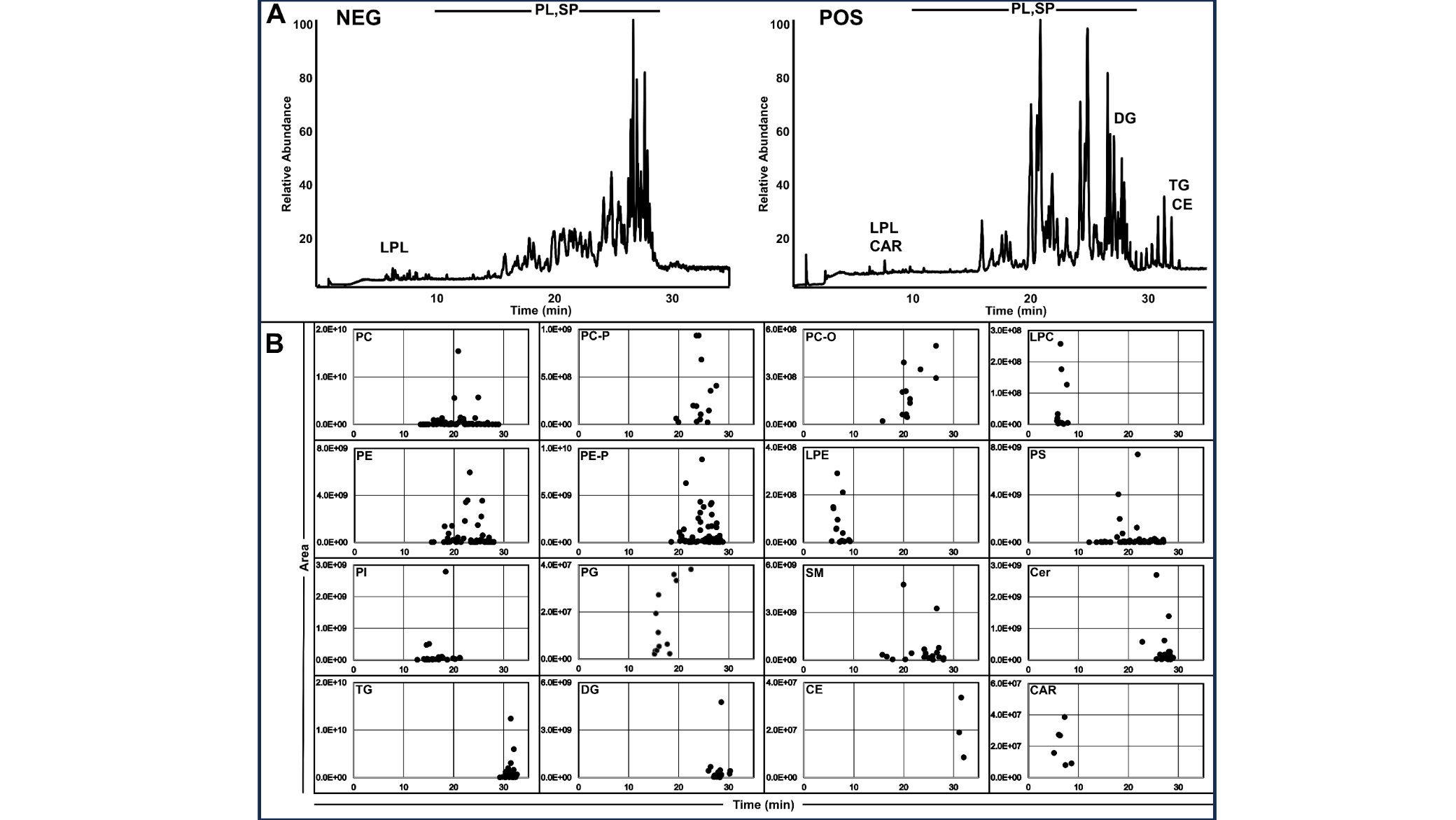


S 2. Brain Extract. A: Chromatogram of 1 mg/mL bovine brain extract in negative and positive mode using 30 min gradients. B: Abundance and elution time of lipid molecular species confirmed by fragmentation pattern and separated by lipid class. Each point represents one major species.


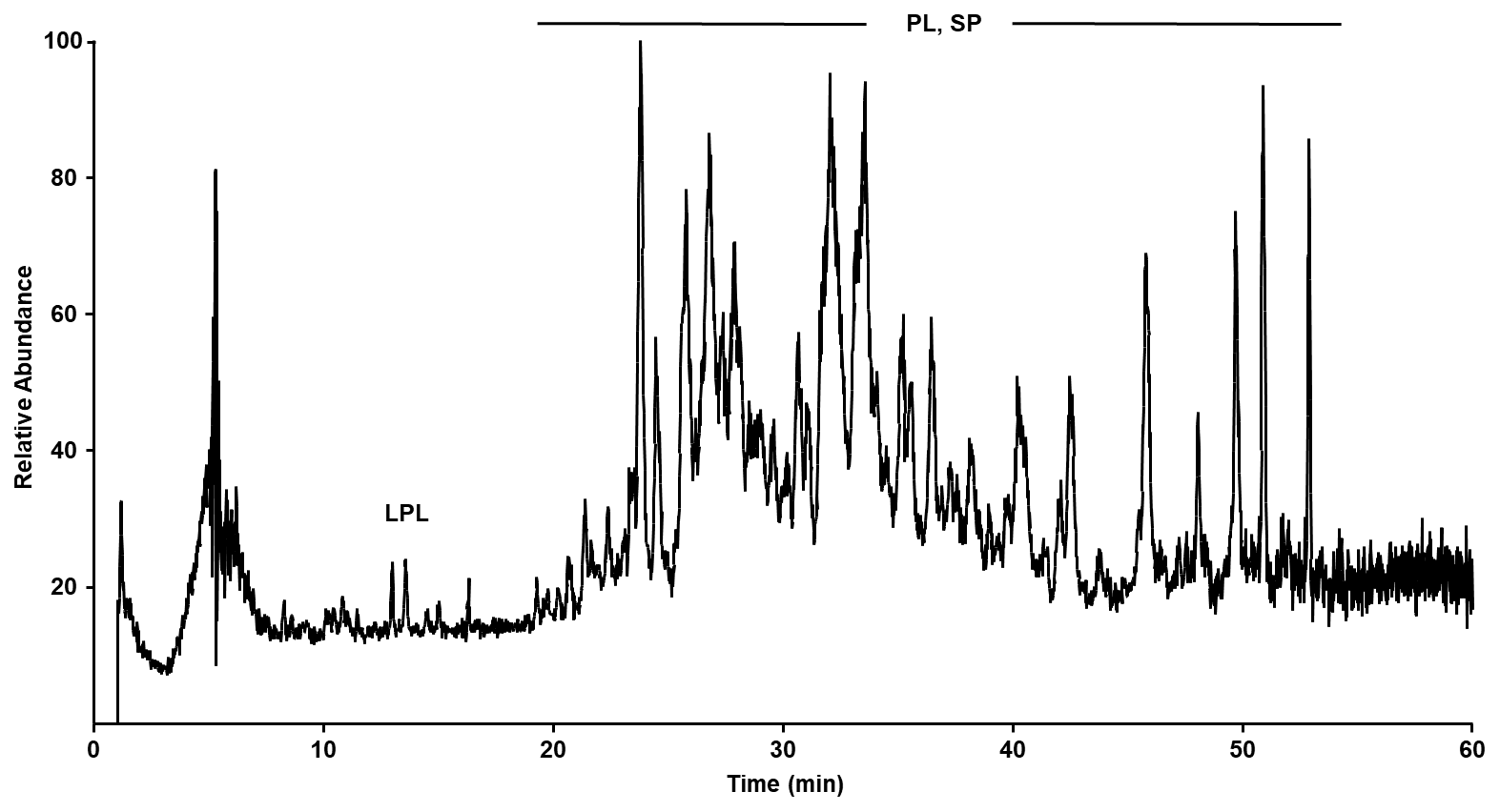


S 3. Chromatogram of RAW cells. Chromatogram of 100 µL of RAW 264.7 cells (1.8 mg protein) homogenized into 500 µL 10% methanol in negative mode using the 60 minutes gradient.


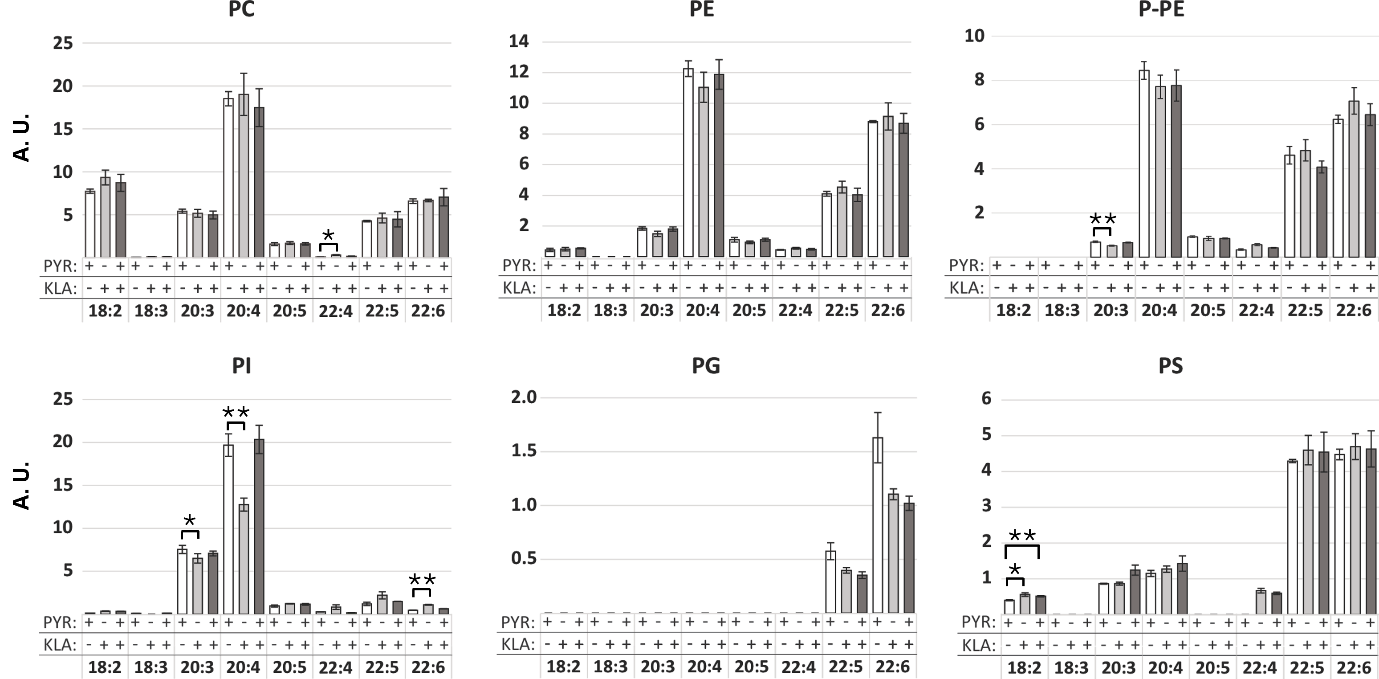


S 4. Full Data Set of cPLA_2_ Assay in RAW Cells. cPLA_2_ activity toward major polar groups and their PUFA content in RAW cells. Polar group phospholipid PUFA composition in the presence or absence of cPLA_2_ inhibitor (PYR) and/or KLA for 24 hours. Cells treated with PYR alone (white), KLA alone (light gray), PYR pre-treatment followed by 24h KLA stimulation (dark gray). The release of FA in the presence of KLA is visible in PI/20:4 and in PG/22:6. However, only the PI/20:4 release is blocked by the cPLA_2_ inhibitor. Statistical significance is indicated by the asterisk (*) and is otherwise not significant.


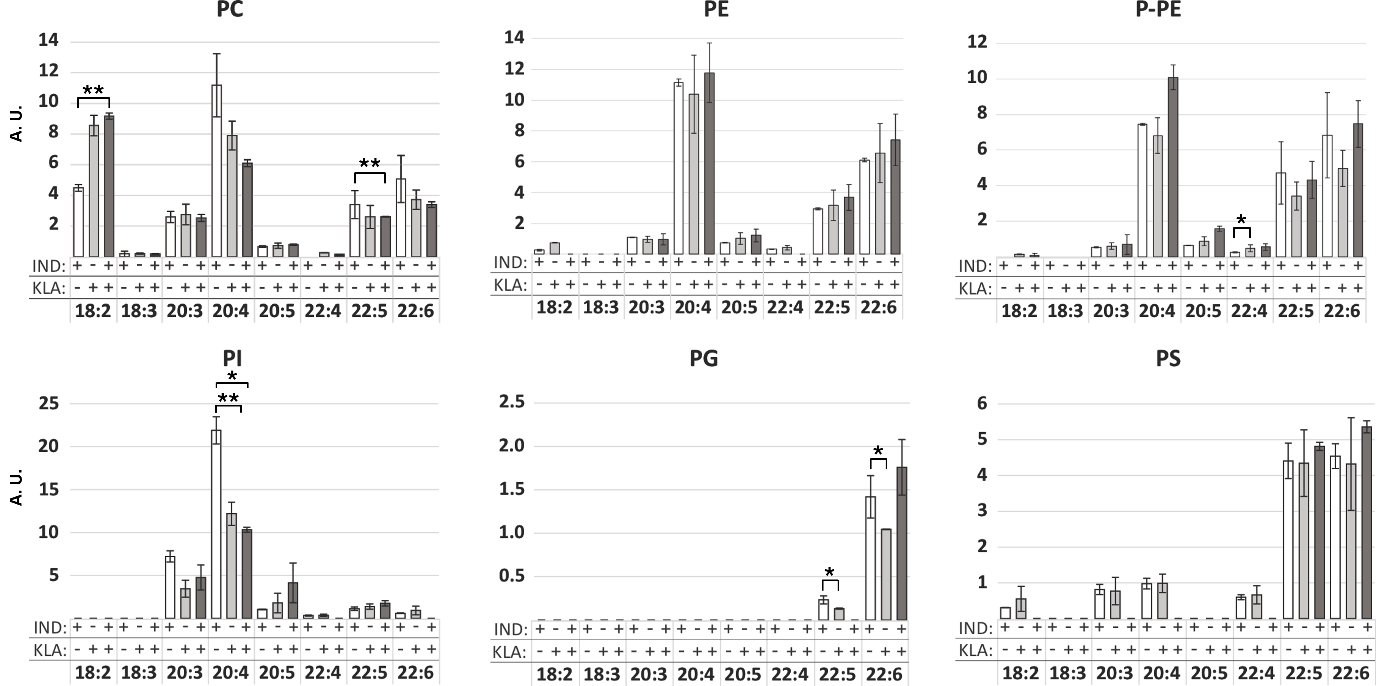


S 5. Full Data Set of sPLA_2_ Assay in RAW Cells. sPLA_2_ activity toward major polar groups and their PUFA content in RAW cells. Polar group phospholipid PUFA composition in the presence or absence of sPLA_2_ inhibitor (IND) and/or KLA for 24 hours. Cells treated with IND alone (white), KLA alone (light gray), IND pre-treatment followed by 24h KLA stimulation (dark gray). The release of FA in the presence of KLA is visible in PI/20:4 and in PG/22:6. However, only the PG/22:6 release is blocked by the sPLA_2_ inhibitor. Statistical significance is indicated by the asterisk (*) and is otherwise not significant.


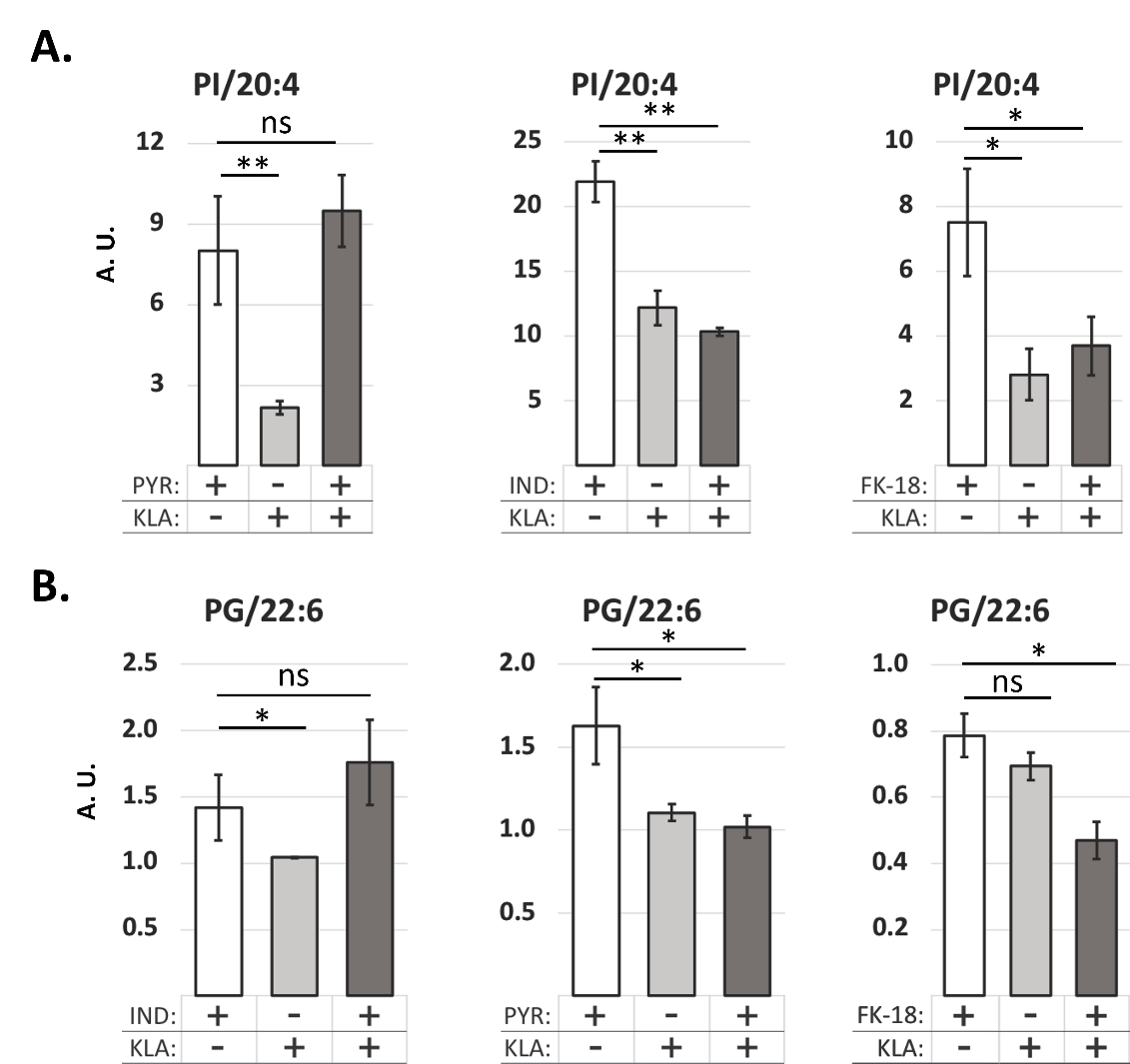


S 6. cPLA_2_ and sPLA_2_ Activity Is Blocked Only with Its Own Inhibitor. A: cPLA_2_ activity toward sn-2 20:4 PI. Control (white), 24h KLA stimulation (light gray), cPLA_2_ inhibitor (PYR) (black), sPLA_2_ inhibitor (IND) or iPLA_2_ inhibitor (FK-18) pretreatment followed by 24h KLA stimulation (dark gray), from left to right, respectively. B: sPLA_2_ activity toward sn-2 22:6 PG. Control (white), 24h KLA stimulation (light gray), sPLA_2_ inhibitor (IND) (black), cPLA_2_ inhibitor (PYR) or iPLA_2_ inhibitor (FK-18) pretreatment followed by 24h KLA stimulation (dark gray), from left to right respectively.


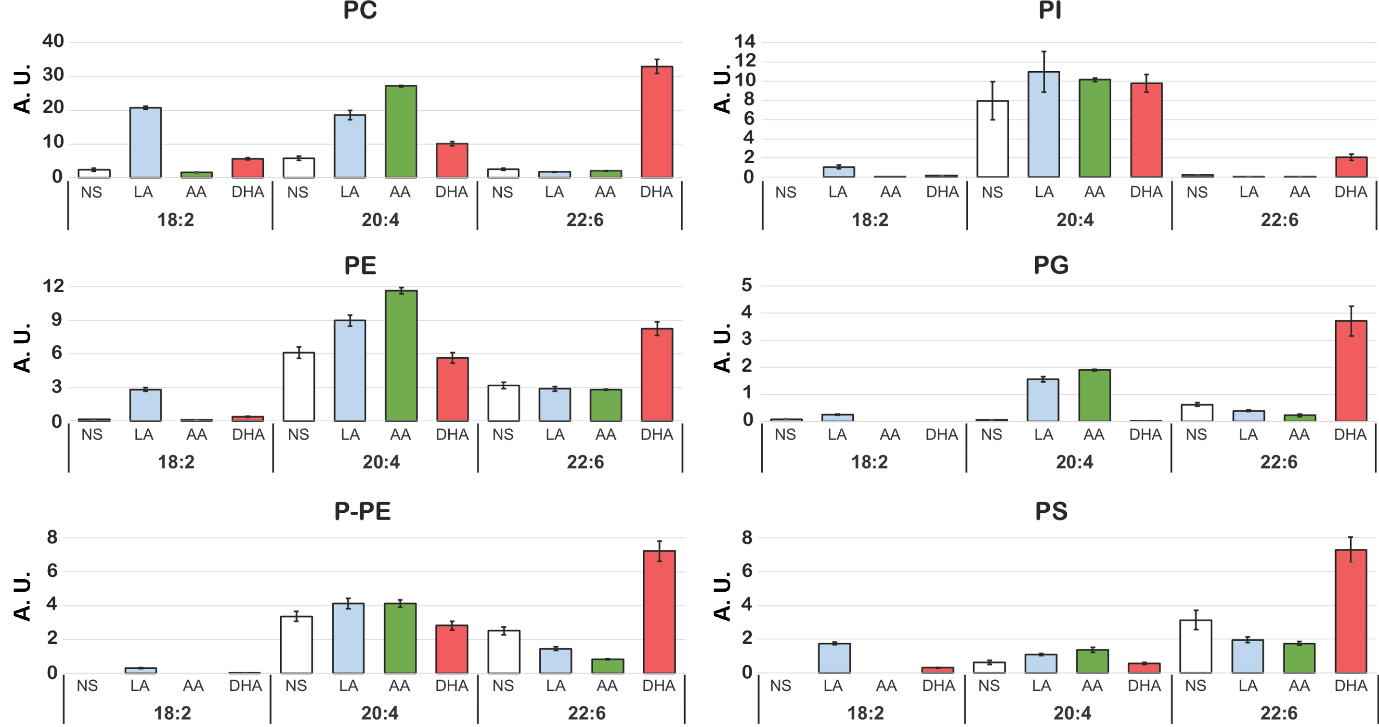


S 7. Polar Group Phospholipid PUFA Composition in RAW Cells Before and After PUFA Supplementation. Cells were supplemented with nothing (white), 18:2 (blue), 20:4 (green) or 22:6 (red). Supplementation with 18:2 shows increase of 18:2 in PC as well as its elongated product 20:4. Supplementation with 20:4 does not significantly increase the 20:4 levels in PI. Supplementation with 22:6 shows dramatic increase of 22:6 across all major polar groups, including PG.


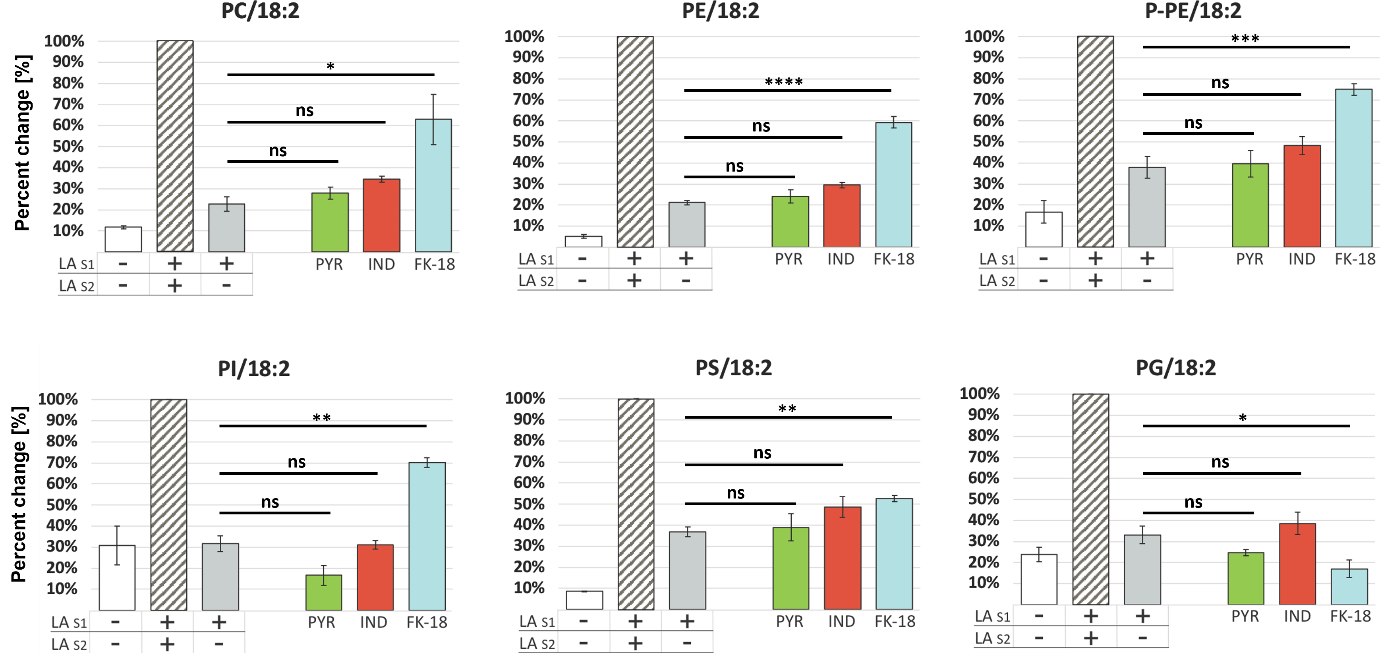


S 8. iPLA_2_ Activity Assay with cPLA_2_ and sPLA_2_ Inhibitors as Controls. iPLA_2_ activity toward sn-2 18:2 PC, PE, P-PE, PS, PI, PG and PS. Negative control (white), positive control (stripes) – cells supplemented with 18:2 FA for 24 hours, iPLA_2_ activity measured for 24 hours (light gray), cPLA_2_ inhibitor (PYR - green), sPLA_2_ inhibitor (IND - red) and iPLA_2_ inhibitor (FK-18) for 24 hours (blue). PYR and IND do not significantly inhibit 18:2 release from any polar headgroup, while FK-18 does significantly inhibit 18:2 release from all polar groups, except PG. (Note, for transparency, the PG data is shown, but the absolute amount of 18:2 in PG after supplementation is minimal and therefore the result for FK-18 inhibition of iPLA_2_ activity toward 18:2 PG is not significant. Data normalized to vehicle treatment for each polar group and displayed as percentage change.


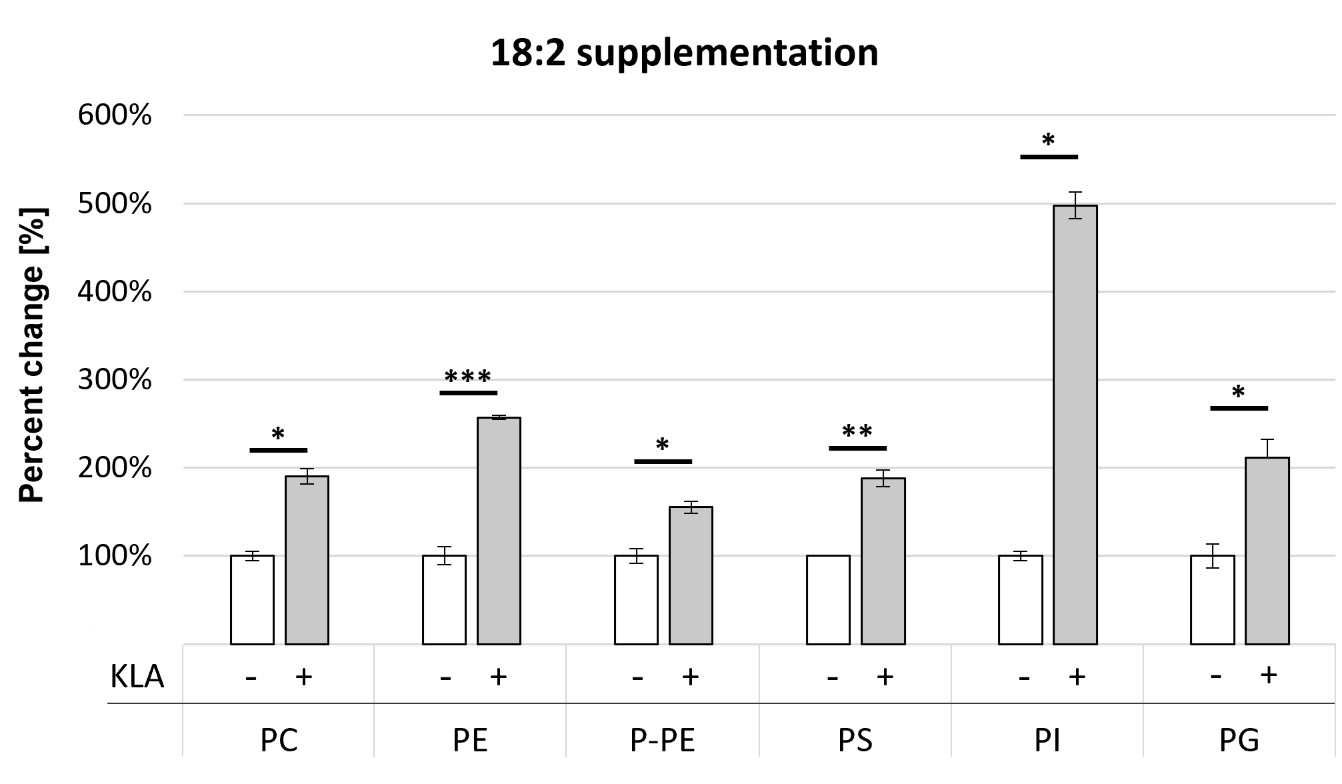


S9. 18:2 as a Substrate for cPLA_2_ and sPLA_2_. Cells supplemented with 18:2 and treated either with media (white) or KLA (gray). Elevated levels of 18:2 across all major polar groups upon KLA stimulation over 24 h suggest that cPLA_2_ is acting to release 20:4 from PI 20:4, which is replaced by the abundance of 18:2 in the media from supplementation. Likewise, sPLA_2_ is releasing 22:6 from PG and is also replenished by the abundance of 18:2. While sPLA_2_ may act on the 18:2 PG, there is so little of it in PG, even after supplementation, it’s activity toward 18:2 PG cannot be assessed. Like in Fig. S5, even if sPLA_2_ is active toward 18:2, the data is not significant, so we are not able to measure cPLA_2_ or sPLA_2_ ex vivo activity toward 18:2 PI or PG in this assay. Data normalized to vehicle treatment for each polar group and displayed as percentage change.

***
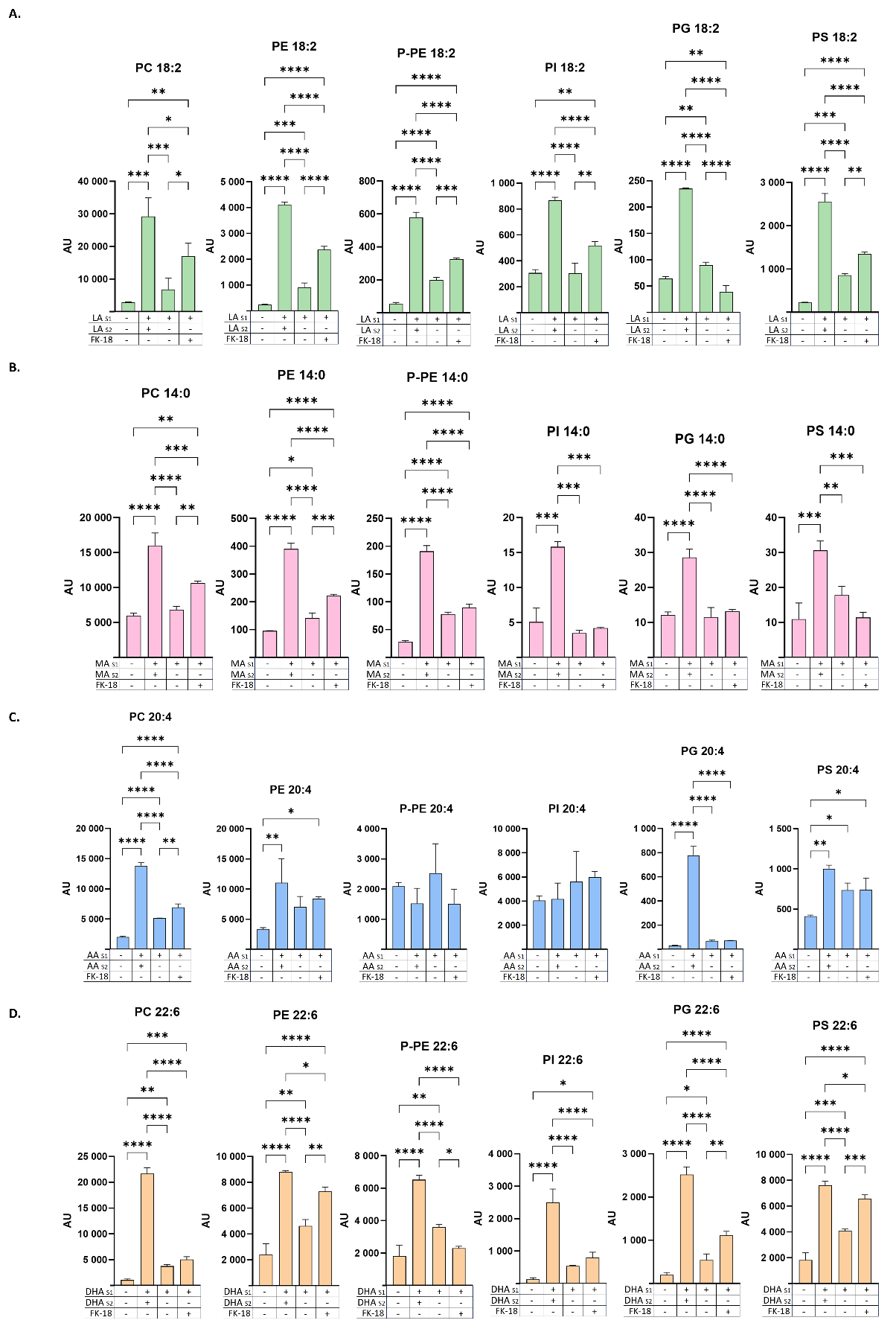
S 10. Full Data Set for iPLA_2_ Assay.*** ***A:*** *iPLA_2_ activity toward supplemented sn-2 18:2 PC, PE, P-PE, PI, PS and PG.* ***B:*** *iPLA_2_ activity toward supplemented sn-2 14:0 PC, PE, P-PE, PI, PS and PG.* ***C:*** *iPLA_2_ activity toward supplemented sn-2 20:4 PC, PE, P-PE, PI, PS and PG.* ***D:*** *iPLA_2_ activity toward supplemented sn-2 22:6 PC, PE, P-PE, PI, PS and PG.* *Statistical analysis was performed using the ordinary one-way ANOVA with Tukey’s multiple comparison’s test. For simplicity, a not-significant change is not labeled.*


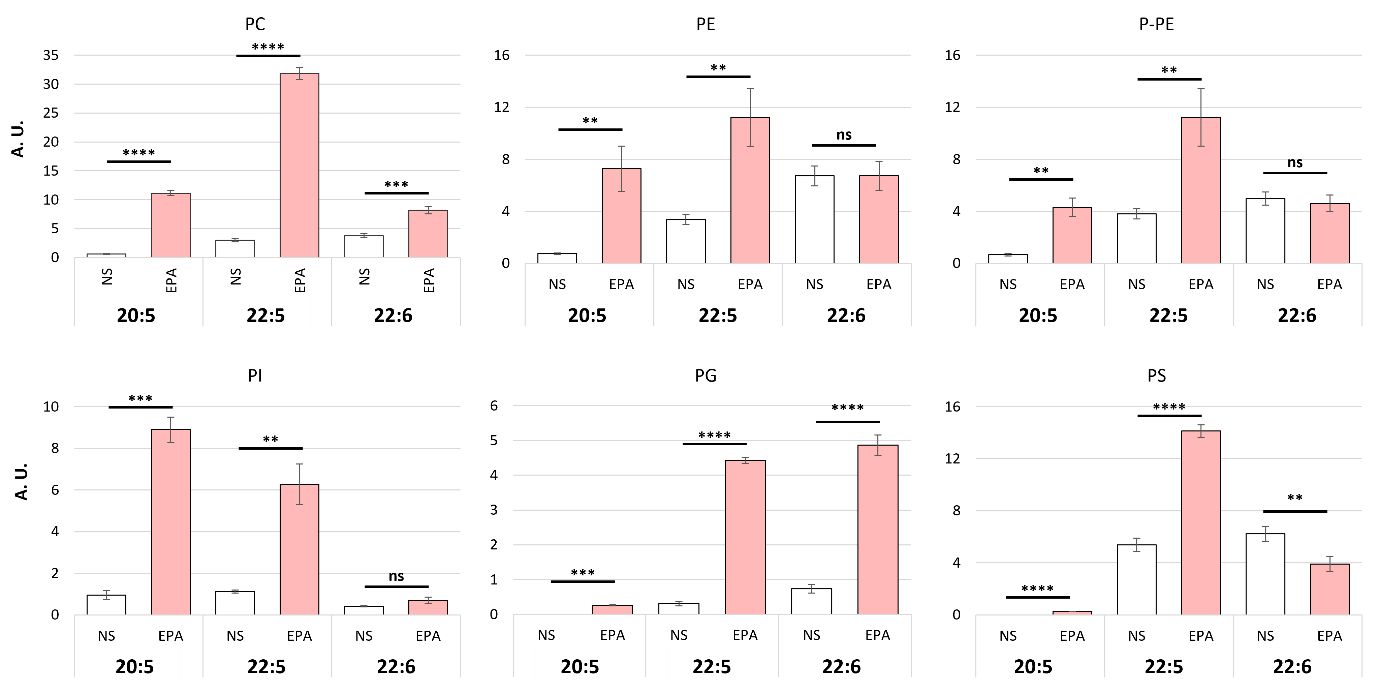


S 11. Supplementation with 20:5 and Its Elongation to 22:5 and 22:6 in RAW 264.7 Cells. Cells were supplemented with media (white) or 20:5 (pink). Supplementation with 20:5 shows a similar increase of 20:5 as well as its elongated product 22:5 and 22:6 in PC, PE and P-PE. However, PI mostly contains 20:5 and 22:5, while in PS and PG the 20:5 levels are close to zero.
